# A multimodal physiological-psychological data-driven study on differentiation in miners’ job burnout and risk preferences

**DOI:** 10.3389/fpsyg.2024.1438772

**Published:** 2024-10-15

**Authors:** Fangyuan Tian, Weishuai Qiu, Hongxia Li, Ziyi Zhao

**Affiliations:** ^1^Management School, Xi'an University of Science and Technology, Xi'an, China; ^2^Xi'an Key Laboratory of Human Factors and Intelligence for Emergency Safety, Xi'an, China

**Keywords:** personality traits, job burnout, emotion regulation strategies, mediating moderation, risk preference

## Abstract

**Background:**

Existing research indicates that the personality traits of miners influence their emotional regulation strategies, and these strategies in turn affect their performance in work fatigue. However, whether there is an intermediary or moderating role among these factors remains unclear. Additionally, while some studies suggest an increased likelihood of unsafe behavior among miners following fatigue, physiological data concerning their cognition remains incomplete and requires further exploration. This study aims to explore the mediating and moderating effects of emotional regulation strategies among miners regarding their personality traits and work fatigue, and to expand understanding of the cognitive and physiological data related to miners’ risk decision-making following fatigue.

**Method:**

Fifty adult miners (Mage = 25, aged 18–40, 100% male) were selected as participants. Emotional regulation tendency, significant levels of personality traits based on the Big Five Personality Traits questionnaire, and the three-dimensional levels of work fatigue were measured using emotional regulation strategy scale, Big Five Personality Traits questionnaire, and work fatigue scale, respectively. The eye–brain consistency hypothesis posits that eye movement trajectories and fixation points reflect the brain’s cognitive processes and focus. Therefore, combining eye-tracking experiments, miners’ preferences in risk decision-making were further measured.

**Results:**

Expressive suppression strategies mediated between conscientiousness and depersonalization; expressive suppression strategies moderated between agreeableness and emotional exhaustion. In eye-tracking physiological experiments, significant differences were found in eye movement data among miners with varying levels of emotional exhaustion.

**Conclusion:**

Preferences in emotional regulation strategies play mediating and moderating roles between miners’ Big Five Personality Traits and work fatigue. The levels and dimensions of work fatigue are influenced not only by personality traits but also by individual tendencies in emotional regulation strategies, which significantly affect performance in risk decision-making. The findings of this study can further enrich theories related to work fatigue among miners and provide insights for personalized safety management in mining.

## 1 Introduction

In China, coal is the primary source of energy. According to incomplete statistics, from January 1 to December 28, 2023, there were 99 coal mine safety accidents nationwide, resulting in 277 deaths and 73 injuries. Statistics on the causes of these accidents indicate that over 80% were due to human factors.^1^

The coal mining production system is complex, and the production environment is constantly changing. Both frontline miners and management-level miners are integral to coal production, so the safety of coal mining enterprises is closely related to the working conditions of miners. The working environment for miners differs from other industries; they need to complete tasks such as line maintenance and gas concentration monitoring in high-temperature and high-humidity conditions. Currently, most coal mines operate on a three-shift rotation system, with each shift lasting over 8 h. Therefore, the harsh working environment and heavy workload make it easy for miners to develop negative emotions at work (Li et al., 2022). If emotion regulation strategies are not properly chosen, the accumulation of negative emotions over time can adversely affect miners, leading to problems in their working condition and significantly increasing the risk of safety accidents (Yang et al., 2020). Frontline miners are the last line of defense for coal mine safety. Only by maintaining a clear and safe state can they minimize the occurrence of safety accidents, thereby enabling the organization to effectively respond to emergencies and continuously provide driving force for the organization’s development.

When facing negative emotions, miners, as individuals, have different personality traits across various dimensions. These differentiated personality traits lead miners to choose different emotion regulation strategies when dealing with negative emotions (Gracanin et al., 2020). Different emotion regulation strategies influence the degree to which miners can alleviate negative emotions. When there is a conflict between one’s personality traits and the chosen emotion regulation strategy, it not only fails to effectively alleviate negative emotions but also exacerbates them, leading to adverse physiological or psychological states in miners. Adverse physiological or psychological states refer to conditions that may lead to unsafe behaviors. These states are potential causative factors and are important links in the causation pathway.

Job burnout is a state in which an individual develops a negative perception of themselves and their external environment due to prolonged engagement in unreasonable work arrangements. It includes three dimensions: emotional exhaustion, low personal accomplishment, and depersonalization (Edu-Valsania et al., 2022). Miners’ emotional exhaustion is primarily manifested as low mood and lack of enthusiasm for work; low personal accomplishment is shown by a lack of achievement and satisfaction in their work; and depersonalization is reflected in a negative attitude toward work and a low sense of responsibility. Previous studies have shown that individuals experiencing long-term job burnout are more likely to exhibit unsafe psychological or behavioral states, such as impulsive risk-taking, depression, and job dissatisfaction, compared to those without job burnout (Platt et al., 2012). Therefore, this research posits that these negative states of job burnout may affect miners’ thoughts and behaviors, significantly increasing the likelihood of safety accidents.

In summary, this study has found that the majority of coal mine safety accidents are caused by human factors. Among these human factors, emotions act as an intermediary variable, which is influenced by personality traits and, in turn, affects job burnout and safety risks. However, research on the mechanisms underlying this pathway is relatively sparse, and the behavioral characteristics within this mechanism need further exploration. Therefore, this study aims to elucidate the relationships among personality traits, emotional regulation strategies, and job burnout, and to further reveal the safety risk decision-making characteristics within this pathway through physiological experiments. Furthermore, it seeks to assist coal mine management in identifying latent human-related hazards, providing a foundation for developing effective interventions, thereby helping to reduce the incidence of safety accidents and improve miners’ work efficiency and safety standards. This is the significance of the study.

## 2 Literature review and research hypotheses

### 2.1 S-O-R model

The “SOR” model, proposed by Mehrabian and Russell based on environmental psychology, posits that stimuli in the environment influence an individual’s emotional state, thereby affecting their behavior (Mehrabian and Russell, 1974). This model has been most widely applied in consumer research. As research has expanded, the model has been integrated into other fields: Chen Weike introduced the model to the study of construction workers’ safety behavior, exploring the influence of emotional intelligence and embodied cognition on safety behavior, and confirmed that the SOR theory is an effective predictive theory (Chen and Liu, 2021); Li Jing et al. analyzed the violation behavior of coal miners based on this model and confirmed it as an effective theory to explain such behavior (Li et al., 2023); additionally, other scholars have applied the model to studies on construction workers (Feng and Liu, 2024). Therefore, the “SOR” model can be applied to research predicting individual safety behavior. In the “SOR” model, “S” represents external stimuli, “O” represents the organism, and “R” represents the response behavior, which aligns with the logic of this study. There is literature discussing the relationship between job stressors and job stress (Szerencsi et al., 2012) which can also be understood as a short-term or immediate stress response or change in mood, especially if the stress is caused by immediate demands at work, tight deadlines, or other features of the work environment that require an immediate response. In addition, there are studies that delve into the relationship between personality traits and job burnout based on the SOR model of personality and emotion (Hendrikx et al., 2024). However, few studies have included emotion regulation strategies as part of the body into this model, and its pathway mechanism under this model remains to be explored.

**Figure 1:**
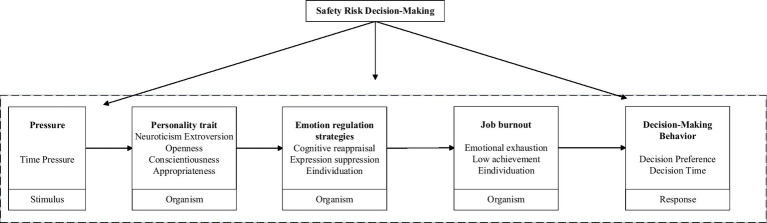
Safety risk decision-making “SOR” model.

In this research, if individuals need to complete a large number of tasks within deadlines or a short period of time, time constraints become a source of stress, which can be denoted as stressor “S.” This stressor can influence individuals’ personality traits. Miners may experience emotions such as tension, anxiety, or excitement when facing external pressure. After experiencing these emotions, miners with different personalities may choose different emotional regulation strategies. Therefore, both personality traits and emotional regulation strategies manifest as internal psychological characteristics of individuals and can be considered as organism variables “O.” Under the influence of emotional regulation strategies, emotions such as tension, anxiety, and excitement can be suppressed or reappraised. These internal organismic emotions affect miners’ decision-making behaviors, demonstrating tendencies toward risk-taking or conservatism. Thus, risk decision-making can be seen as an organismic reflection “R.” So in this research, time pressure serves as the external stimulus “S,” personality traits and emotion regulation strategies as the organism “O,” and job burnout and safety risk decision-making as the response “R,” constructing the following safety risk decision-making SOR model, as shown in Figure 1.

### 2.2 Personality traits and job burnout

Individual personality traits are closely related to the risk of job burnout. Currently, many scholars have conducted detailed research on the relationship between the Big Five personality traits and job burnout: Giacomo Angelini found through a systematic literature review that higher neuroticism and lower agreeableness, conscientiousness, extraversion, and openness are significantly correlated with job burnout (Angelini, 2023). Renzo Bianchi found through questionnaire surveys that personality trait indicators seem to play a more important role in job burnout research (Bianchi, 2018). Anna Bashkirova et al. found through adaptive network modeling that neuroticism is positively correlated with emotional exhaustion, while extraversion and conscientiousness show varying relationships with dimensions of job burnout (Bashkirova et al., 2023). Furthermore, current research on personality traits and job burnout, particularly in the teaching profession (Hu and Zhao, 2019), shows significant correlations between neuroticism, conscientiousness, agreeableness, and depersonalization, whereas extraversion and openness are significantly associated with emotional exhaustion and low personal accomplishment. In addition, research on individuals in professions such as doctors (Liang et al., 2018) and public officials (Feng et al., 2022; Wu et al., 2024) shows significant correlations between personality traits and various dimensions of job burnout, though the relationships between different personality traits and dimensions of job burnout vary and require further exploration. Therefore, personality traits have a significant predictive effect on job burnout, but the mechanisms through which the Big Five personality traits lead to differences in job burnout manifestations are still not well understood, and further research in this field remains unclear. Based on the above literature, this study proposes the following hypotheses:

*H1:* Personality traits influence differences in job burnout.

### 2.3 The mediating and moderating roles of emotion regulation strategies

Emotion regulation strategies serve as significant predictors of job burnout. These strategies are defined as the processes through which individuals adjust or express emotions and experiences using cognitive or behavioral strategies. The most commonly utilized emotion regulation strategies include cognitive reappraisal and expressive suppression. Cognitive reappraisal involves the tendency to think about situations differently to alter emotional impact, often associated with positive emotions. On the other hand, expressive suppression refers to attempts to suppress thoughts and emotions associated with situations and emotional expression, which may potentially exacerbate negative emotions. Currently, scholars have conducted research on the differences in emotion regulation strategies and the manifestation of job burnout. Cheng Hongling explored the impact of emotion regulation strategies on job burnout and found that these strategies serve as significant predictors of job burnout (Cheng and Chen, 2010). They highlighted that using strategies such as suppressing negative emotions or engaging in surface acting are key factors leading to job burnout. Goussinsky found that the main reasons for nursing staff’s job burnout are work attitudes and surface acting, suggesting that emotion regulation strategies can influence the level of job burnout among nurses (Goussinsky and Livne, 2016). Bamonti studied the relationship between job burnout levels and emotion regulation strategies among elderly care workers, revealing a closer relationship between personality disintegration dimensions and emotion regulation strategies (Bamonti et al., 2022). Shen Yong et al. conducted a survey using a professional burnout scale and found that dental interns generally experience varying degrees of professional burnout (Shen et al., 2021). They found a close relationship between expressive suppression and cognitive reappraisal abilities and the level of professional burnout among dental interns. Michinov conducted a cross-sectional study on French firefighters, utilizing conflict management styles, emotional intelligence, and a burnout scale to measure job burnout (Michinov, 2022). They found that firefighters regulate their emotions to reduce emotional exhaustion in response to specific job emotional demands. Thomas studied the effects of self-differentiation strategies and job satisfaction on individual and job burnout, revealing that self-differentiation strategies provide resources for pastors to combat personal burnout experiences, while job satisfaction protects pastors from the impact of job burnout (Frederick et al., 2023). In summary, current research indicates that emotion regulation strategies play a significant role in the level and manifestation differences of job burnout. However, there is limited research on the pathways through which emotion regulation strategies affect the dimensions of job burnout, necessitating further exploration and refinement of related mechanisms.

Personality traits and emotion regulation strategies are related. Ciydem et al. investigated the impact of personality traits and emotion regulation strategies on adolescents’ high-risk behaviors, finding a certain linear relationship between the two (Ciydem et al., 2024). Kluwe-Schiavon et al. found that difficulties in emotion regulation and high levels of neuroticism might be potential risk factors for psychiatry during the COVID-19 pandemic, indicating a significant correlation between the two (Bruno et al., 2023). Kanj and Hallit explored the association between childhood emotional abuse and borderline personality disorder in Lebanese adults, with the mediating role of emotion regulation difficulties, suggesting that training in emotion regulation strategies may help prevent the development of borderline personality disorder and promote treatment (Kanj et al., 2023). Sun Juncai et al. based on the action control theory of emotion regulation, found through experimental methods that individuals with high agreeableness traits have implicit advantages in the action control of emotion regulation, providing insights into how agreeableness affects individuals’ emotion regulation (Sun et al., 2019). Zhao Xin et al. explored the relationship between adolescents’ personality traits and social anxiety, analyzing the mediating role of emotion regulation methods. They found that the frequency of cognitive reappraisal is significantly negatively correlated with social anxiety, while expressive suppression is significantly positively correlated with it. Moreover, cognitive reappraisal and expressive suppression play partial or complete mediating roles between personality traits and social anxiety, providing certain insights for the psychological intervention of adolescent social anxiety (Zhao et al., 2014). To explore the impact of teachers’ self-compassion, emotion regulation, and emotional labor measurement on teachers’ resilience in the context of teaching English as a foreign language, Hu used confirmatory factor analysis to assess teachers’ characteristics in these dimensions, ultimately finding that improving self-compassion, emotion regulation, and emotional labor strategies can enhance teachers’ psychological resilience (Hu, 2023). Therefore, there is a correlation between personality traits and emotion regulation strategies. Based on the above literature, this study proposes the following hypotheses:

*H2:* Emotion regulation strategies mediate the relationship between personality traits and job burnout performance differences.

Emotion regulation strategies play a significant moderating role in psychological traits and behavioral performance. Recent studies on the moderating effects of emotion regulation strategies have shown that cognitive reappraisal and expressive suppression significantly impact college students’ cognitive rumination and aggression (Zhao and Zhang, 2019). In the context of work burnout, Cheng Hongling et al. found that suppressing negative emotions, specifically expressive suppression, is a key factor leading to burnout, while individual or organizational factors such as job autonomy can moderate the relationship between emotion regulation strategies and burnout (Cheng and Chen, 2010). In specific professions like emergency nursing, Liao Liwen et al. found that emotion regulation strategies significantly moderate the relationship between psychological resilience and coping methods, reducing nurses’ stress resilience and mitigating negative impacts on individuals and work, as well as burnout (Liao and Wang, 2023). In a study on college students’ aggression, Zhang Shanshan et al. found that cognitive reappraisal significantly moderates the relationship between emotional rumination and aggression (Zhang et al., 2024). To investigate the effects of emotion regulation strategies on cognition and behavior in social contexts, Guo Xiaodong et al. validated through surveys that cognitive reappraisal and expressive suppression have a moderating effect on empathy and negative emotional cognition (Guo et al., 2023).

Therefore, after examining the above literature, this study finds that emotion regulation strategies not only mediate but also moderate the relationship between traits and behaviors. Additionally, the research on the moderating effects of emotion regulation strategies between personality traits and work burnout is limited. Thus, to further explore the role of emotion regulation strategies, this study proposes the following hypothesis:

*H3:* Emotion regulation strategies moderate the relationship between personality traits and job burnout performance differences.

The above analysis indicates that many scholars have explored the relationships between personality traits, emotion regulation strategies, and job burnout in various professions such as teachers, doctors, and nurses. However, few studies have examined the path mechanisms and effects of emotion regulation strategies on the differences in job burnout expressions among individuals with different Big Five personality traits. Current research on high-risk occupational groups such as miners is still limited, and no scholars have investigated the impact of emotion regulation strategies on the Big Five personality traits and job burnout in this specific group. Therefore, this study aims to explore the mediating and moderating path mechanisms and effects of emotion regulation strategies on the differences in job burnout expressions among miners with different personality traits. Additionally, the S-O-R model is one of the foundations of modern cognitive psychology, which specifically explains the predictive impact of environmental characteristics on individual emotional responses and subsequent behavior. According to this theory, external environmental pressure stimulates the organismic characteristics of miners’ personality traits and emotion regulation strategies, resulting in different expressions of job burnout, which ultimately affect their safety risk decision-making behavior. In summary, this study will be based on the S-O-R model, focusing on the path mechanisms of personality traits-emotion regulation strategies-job burnout differences through interviews, questionnaires, and scales. It will quantitatively analyze whether different personality traits lead to different job burnout expressions through varying emotion regulation strategies, and explore the internal path mechanisms, aiming to provide new methods and insights for the intervention and management of miners’ job burnout.

**Figure 2:**
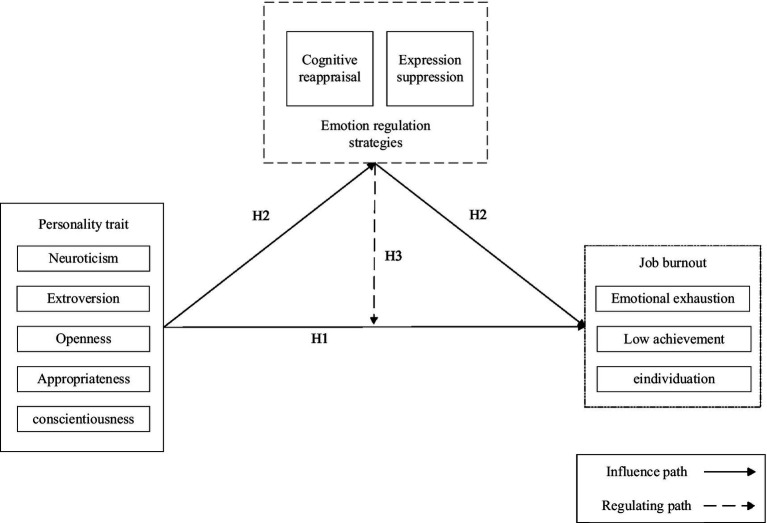
Conceptual model of the differential effects of miners’ personality traits on job burnout performance.

In summary, based on the above hypothesized relationships, this paper constructs a conceptual model of the differences in job burnout performance among miners based on personality traits, as shown in Figure 2.

## 3 Data sources and research methods

### 3.1 Sample selection and data collection

This study employed a random sampling method to conduct field surveys and questionnaires among miners at a coal mine in Shaanxi, China. Given that coal mining work is generally conducted in groups of five, a total of 10 groups, or 50 miners, were randomly selected. After excluding unmatched, incomplete, or multiple responses, 48 valid questionnaires were obtained, yielding a 95% effective response rate. Due to the nature of the mining profession, all 48 miners surveyed were male; 23 were under 25 years old (47.9%), 20 were between 25 and 35 years old (41.7%), and 5 were over 35 years old (10.4%). The demographic information of the participating miners is shown in the table below.

### 3.2 Variable measurement method

#### 3.2.1 Measurement of personality traits, emotion regulation strategies, and job burnout levels

The scales used in this study are mature scales published in authoritative academic journals in both Chinese and English (Luo et al., 2019; Chen et al., 2020; Zhao et al., 2023). The Big Five personality scale adopts a Likert 5-point scale (1–5 indicating “strongly disagree” to “strongly agree”), consisting of five dimensions: neuroticism, extraversion, openness, agreeableness, and conscientiousness. The emotion regulation strategy scale adopts the ERQ questionnaire by Gross et al., using a Likert 7-point scale (1–7 representing “strongly agree” to “strongly disagree”), including two measurement dimensions: cognitive reappraisal and expressive suppression. The job burnout scale uses the internationally recognized Mbi-Gs questionnaire for job burnout measurement, using a Likert 7-point scale (0–6 representing “never” to “very frequently”), consisting of three dimensions: emotional exhaustion, low sense of accomplishment, and depersonalization. Some of the questions are shown in Table 1.

**Table 1:** Latent variables measurement item.

Latent variables	Measurement items (part)
Personality trait (PT)	I’m not a worrier—Neuroticism (PT1)
	I like having a lot of people with me—Extroversion (PT2)
	I like to indulge in fantasies and daydreams and explore all possibilities and let them spread and develop—Openness (PT3)
	I try to be polite to everyone I meet—Appropriateness (PT4)
	I put my things away and keep them clean—conscientiousness (PT5)
	…
Emotion regulation strategies (ERS)	When I want to feel something positive (like joy or joy), I change the way I think about things—Cognitive reappraisal (ERS1)
	I do not show my emotions—Expression suppression (ERS2)
	…
Job burnout (JB)	The work wears me out—Emotional exhaustion (JB1)
	I’ve accomplished a lot of good things at work—Low achievement (JB2)
	I do not care what happened to my colleague—Deindividuation (JB3)
	…

#### 3.2.2 Eye tracking technology

According to the hypothesis of oculomotor cognition, changes in eye trajectories and fixation points can reflect an individual’s level of interest in objects and their logical thinking process. They can also indicate the mental state of an individual during thinking. Starting from the Immediacy Assumption and the Eyemind Assumption, numerous research data indicate that the gaze duration on certain information generally corresponds to the cognitive processing time of this information. When cognitive processing is more difficult, that is, when cognitive load is higher, gaze duration tends to be correspondingly prolonged. Furthermore, the concept of regions of interest refers to designating a specific area affecting the data as a focal area according to various test task requirements, recording all eye movement data of the subjects in this area.^2^ This study used a Tobii Pro Spectrum eye tracker to measure the subjects, selecting eye movement data indicators such as eye movement trajectory maps and eye movement heat maps. Specific measurement indicators and contents are shown in Table 2.

**Table 2:** Eye-tracking measurement indicators and measurement content.

Indicators	Measurement Content
Eye tracking trajectory	Individuals’ gaze positions, gaze durations, and gaze sequences when observing stimulus materials. In eye tracking trajectory graphs, observation time is typically represented by circles of varying diameters. The longer the duration of fixation, the larger the radius of the circle, indicating greater attentional engagement. The sequence of fixations is usually displayed on the circles with numbers, such as 1, 2, 3, indicating the first location observed by the participant, followed by subsequent locations, and so on.
Heatmap of eye movements	The average fixation duration is the mean duration of all fixations within an area of interest. Selecting this metric allows for further analysis of the degree of interest in different decisions among participants with different characteristics.

#### 3.2.3 Measurement of safety decision preferences

**Figure 3:**
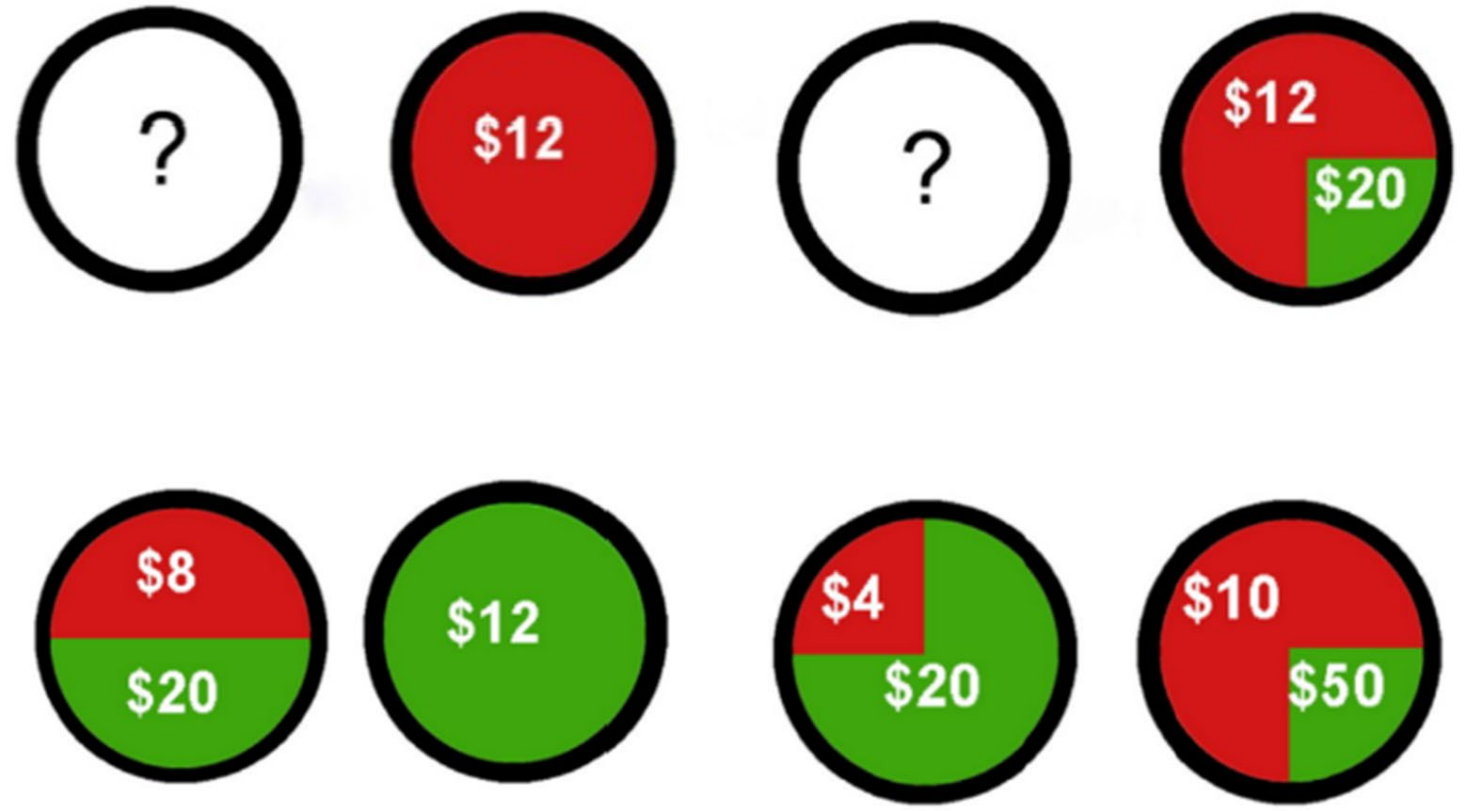
Stimulus material.

The experimental software platform is built on Eprime3.0, using basic programming to design a simplified version of the Wealth Wheel gambling task (Blankenstein et al., 2018). The experiment is divided into a pre-experiment and a formal experiment. The pre-experiment is the Gp group; the formal experiment consists of four types of Wealth Wheel gambling tasks, namely G1, G2, G3, and G4. Within the Gp, G1, G2, G3, and G4 groups, there are four combinations of Wealth Wheel combinations, namely A-R, A-C, R-C, and R-R. Among them, C represents deterministic, A represents ambiguous, and R represents risky. During the formal experiment, a 6-s countdown is introduced to observe miners’ preference for uncertain decision-making under time pressure, as shown in Figure 3.

**Figure 4:**
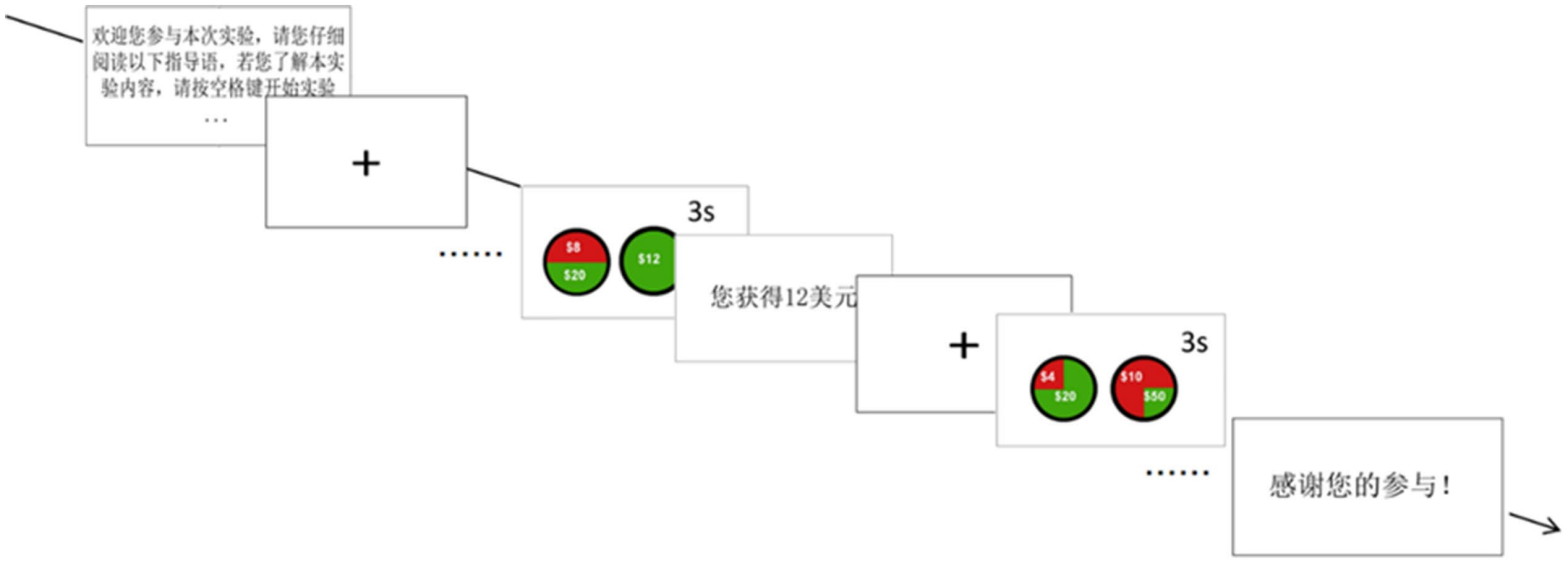
The experimental procedure.

The experimental procedure is shown in Figure 3. The experimental process is shown in Figure 4: Under time pressure, each group of experiments had a decision time of 3,000 ms. If the subject did not make a decision within the allotted time, it automatically skipped to a feedback screen displaying “You received $0.” In this experiment, the decision time was displayed as a countdown in the upper right corner of the interface. After the subject made a choice, a feedback screen displaying “You received X dollars” appeared for 3,000 ms. After this screen, the next round of the experiment began.

Control Variables: Considering other factors that may affect the study results, and referring to previous research, gender, age, and education level of employees were used as control variables to ensure the accuracy and rationality of the tests.

### 3.3 Data analysis methods

In this study, SPSS 27.0 software was used for calculations. Firstly, exploratory factor analysis results showed that the KMO value was greater than 0.8 and the factor loadings of the variables were all greater than 0.8.

Then, the Bartlett’s test of sphericity was significant, and the specific algorithm is as follows in Equations (1–3):

(1) Calculate the Determinant of the Sample Correlation Matrix:

(1) $$\det\left(R\right)=\;|R|$$

where *R* is the sample correlation matrix.

(2) Compute the Bartlett’s Test Statistic (Chi-Square Statistic):

(2) $$\chi^{2}=-\left(n-1-\frac{2p+5}{6}\right)\ln\left(\det\left(R\right)\right)$$

where *n* is the sample size and *p* is the number of variables.

(3) Calculate the Degrees of Freedom:

(3) $$df=\frac{p\left(p-1\right)}{2}$$

Finally, the internal consistency of the questionnaire and scale was assessed using Cronbach’s Alpha to measure reliability in Equations (4–6). Since reverse-scored items can affect the reliability and validity of the questionnaire, they were removed.

(1) Calculate the Variance of Each Item:

(4) $$\sigma_{i}^{2}$$

where *i* represents the *i*-th item.

(2) Calculate the Variance of the Total Scores:

(5) $$\sigma_{T}^{2}$$

(3) Compute Cronbach’s Alpha:

(6) $$\alpha =\frac{k}{k-1}\left(1-\frac{\sum_{i=1}^{k}\sigma_{i}^{2}}{\sigma_{T}^{2}}\right)$$

where *k* is the number of items, $\sigma_{i}^{2}$ is the variance of the *i*-th item, and $\sigma_{T}^{2}$ is the variance of the total scores.

After removal, the Cronbach’s Alpha coefficient of the questionnaire was 0.8. Moreover, the questionnaire used in this study was validated by authoritative academic journals in both Chinese and English, ensuring good reliability and validity.

## 4 Data analysis results

### 4.1 Common method bias

A Harman single-factor test was conducted on all items of the three variables, explaining 71.612% of the total variance, with the largest factor explaining 27.830%, which is below 40%.

### 4.2 Reliability and validity testing

First, this study used SPSS 27.0 software for calculations. The results of exploratory factor analysis showed that the KMO value was greater than 0.7, Bartlett’s test of sphericity was significant, and the factor loadings of the variables were all greater than 0.7. Additionally, the questionnaire used in this study was verified by authoritative academic journals in Chinese and English, indicating good reliability and validity.

### 4.3 Descriptive statistics and correlation analysis

Neuroticism showed a significant positive correlation with the three dimensions of job burnout (*p* < 0.01), openness showed a significant positive correlation with the depersonalization dimension of job burnout (*p* < 0.01), agreeableness showed a significant negative correlation with the low personal accomplishment dimension, emotional exhaustion and low personal accomplishment showed a significant positive correlation with expressive suppression (*p* < 0.01), while depersonalization showed a significant negative correlation with expressive suppression (*p* < 0.01). The study supports H1: Personality traits have a significant impact on differences in individual job burnout. Collinearity tests showed a maximum VIF value of 1.565, well below 5, indicating no collinearity issues. The results are shown in Table 3.

**Table 3:** Descriptive statistics analysis of Big Five personality traits, emotional exhaustion, and emotion regulation strategies.

	*M*	SD	1	2	3	4	5	6	7	8	9	10
1. Neuroticism	35.59	6.32	–									
2. Extroversion	38.38	8.23	−0.173	–								
3. Openness	40.95	4.50	−0.519**	0.062*	–							
4. Appropriateness	38.46	5.08	−0.078	−0.239*	−0.097	–						
5. Conscientiousness	42.79	7.36	−0.587**	0.223	0.263	0.303*	–					
6. Emotional exhaustion	25.23	5.18	0.258**	−0.280*	−0.131	−0.029	−0.226	–				
7. Low achievement	24.66	5.98	0.282**	0.048	−0.228	−0.159*	−0.116	0.376**	–			
8. Deindividuation	34.58	8.05	0.411**	−0.188	−0.316**	0.114	0.034	0.396**	0.313*	–		
9. Cognitive reappraisal	30.45	6.29	0.133*	0.122*	0.103	0.123	0.119	0.131	0.199	−0.066	–	
10. Expression suppression	14.55	3.89	−0.055*	−0.367*	0.079	0.378*	0.216*	0.283**	0.291**	−0.290**	0.074	–

*Indicates *p* < 0.05, **indicates *p* < 0.01.

### 4.4 Mediating regression test

This study used SPSS 27.0 software and applied hierarchical regression and the bootstrap method to test the research hypotheses. First, the raw data is standardized. Secondly, Model 4 in the process procedure of SPSS software was used to analyze the mediating effect, with age and working time as covariates, the five dimensions of the Big Five personality traits as independent variables, the three dimensions of job burnout as dependent variables, and the two dimensions of emotion regulation as mediating variables. In the first step, the five dimensions of the Big Five personality traits were used as independent variables, and the three dimensions of job burnout as dependent variables, for regression analysis. In the second step, the two dimensions of emotion regulation were used as independent variables, and the three dimensions of job burnout as dependent variables, for regression analysis. In the third step, the five dimensions of the Big Five personality traits and the two dimensions of emotion regulation were used as independent variables, and the three dimensions of job burnout as dependent variables, for regression analysis. After filtering out the results with non-significant *p*-values, the following results were obtained in Table 4.

**Table 4:** Mediating regression effects of emotion regulation strategies on personality traits and job burnout.

	Deindividuation	Expression suppression	Deindividuation
Constant	5.687	−18.07	−4.586
Neuroticism	−0.271	0.284	−0.078
Extroversion	0.209	−0.212	0.083
Openness	0.604	0.181	0.728
Appropriateness	0.339	0.265	0.487
Conscientiousness	−0.18	0.306	0.029
Expression suppression			−0.614
*N*	48	48	48
*R* ^2^	0.256	0.311	0.381
Δ*R*^2^	0.136	0.173	0.204
*F*	2.129 (*p* = 0.088)	2.795 (*p* = 0.028*)	2.547 ((*p* = 0.049*)

*Indicates *p* < 0.05.

According to the research methods of Wen and Ye (2014b, 2014a), a bootstrap test needs to be conducted for ab. Here, a refers to the effect coefficient of the independent variable on the mediator variable, b refers to the effect coefficient of the mediator variable on the dependent variable, and ab refers to the product of the interaction terms between the independent variable and the mediator variable, i.e., the effect coefficient of the independent variable on the dependent variable through the mediator variable. After analysis, a meaningful path was identified: conscientiousness → expressive suppression → depersonalization. Both a and b were significant, while c’ was not, indicating a full mediation with an effect size of 100%. Therefore, H2b was validated: the strategy of expressive suppression mediates the relationship between personality traits and job burnout. The following result is obtained in Table 5.

**Table 5:** Analysis of mediation pathways and effect values of expressive suppression.

Pathway	Total effect value	a	b	Mediation effect a*b	Direct effect c’	95% Confidence Interval	
						Lower	Upper
conscientiousness → Expression suppression → deindividuation	−0.180	0.306*	−0.614**	−0.188	0.029	−0.661	−0.000

*Indicates *p* < 0.05, **indicates *p* < 0.01.

### 4.5 Moderation effect test

In addition, for non-significant paths, this study examined the moderation effects of emotion regulation strategy variables in other paths. The first step examined the influence of the independent variable on the dependent variable without considering the interference of moderation variables; the second step involved adding moderation variables on top of the first step, and the third step added the interaction product term between the independent variable and the moderation variable. After testing the moderation effects of personality traits, emotion regulation strategies, and job burnout, it was found that expressive suppression moderates the relationship between agreeableness and emotional exhaustion, as shown in Table 6.

**Table 6:** Testing the moderating effect of expressive suppression on the relationship between agreeableness and emotional exhaustion.

	Model1				Model2				Model3			
	Coefficient	SE	*t*	*p*	Coefficient	SE	*t*	*p*	Coefficient	SE	*t*	*p*
Constant	25.361	6.499	3.902	0.000**	25.67	6.157	4.169	0.000**	−7.91	17.511	−0.453	0.654
Appropriateness	0.009	0.168	0.053	0.958	−0.138	0.172	−0.8	0.429	0.78	0.479	1.626	0.113
Expression suppression					0.365	0.163	2.24	0.032*	2.637	1.126	2.342	0.025*
Appropriateness* Expression suppression									−0.061	0.03	−2.038	0.04*
*R* ^2^	0				0.129				0.226			
△*R*^2^	0				0.129				0.226			
*F*	0.003, *p* = 0.958				5.018, *p* = 0.012*				6.895, *p* = 0.013*			
	Dependent variable: Emotional exhaustion											

*Indicates *p* < 0.05, **indicates *p* < 0.01.

**Figure 5:**
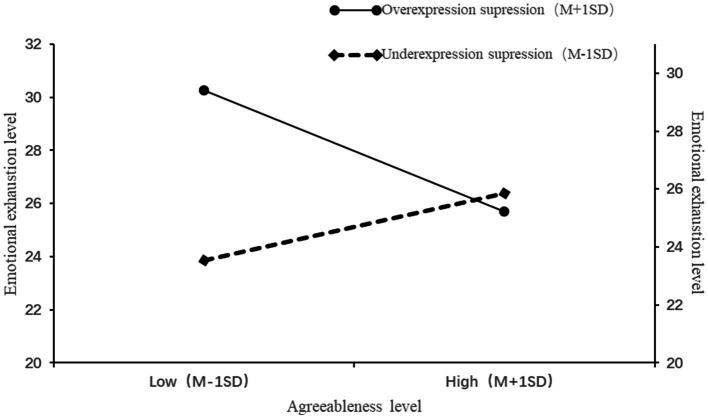
Moderating effect of expressive suppression.

From table and Figure 5, it can be observed that there is a change in the *F*-value from Model 2 to Model 3, and the interaction term in Model 3 is significant, indicating that the expressive suppression strategy moderates the relationship between agreeableness and emotional exhaustion.

### 4.6 Discriminant analysis and eye-tracking experiment data analysis

As shown in table, the classification significance under the three dimensions passed the significance test, indicating good classification effects. The results are shown in Table 7.

**Table 7:** Cluster analysis of the three dimensions of job burnout.

Classification dimension	Clustering		Error		*F*	*p*
	Mean square	Degrees of Freedom	Mean square	Degrees of Freedom		
Emotional exhaustion	212.677	1	21.110	47	10.075	0.003**
Low achievement	506.616	1	22.064	47	22.961	<0.001**
Deindividuation	1,024.164	1	42.209	47	24.264	<0.001**

*Indicates *p* < 0.05, **indicates *p* < 0.01.

The subjects were divided into two groups, high and low job burnout levels, based on the three dimensions. Their behavioral experiment performances were then statistically analyzed. First, the preferences for three types of risks under time pressure were analyzed. Based on the previous research, the dimension of emotional exhaustion was selected as the grouping basis for discriminating the risk selection results. The Fisher function was chosen as the function coefficient. The analysis found that the discriminant analysis results for Class A risk passed the significance test, as shown in Table 8.

**Table 8:** Discriminant analysis of the three types of risk under the emotional exhaustion dimension.

Risk types	Lambda	*F*	Degrees of Freedom 1	Degrees of Freedom 2	*p*	Standardized canonical discriminant function coefficients
A	0.86	4.557	1	46	0.042*	1.138
R	0.991	0.257	1	46	0.616	0.362
C	0.89	3.459	1	46	0.073	–

*Indicates *p* < 0.05.

**Figure 6:**
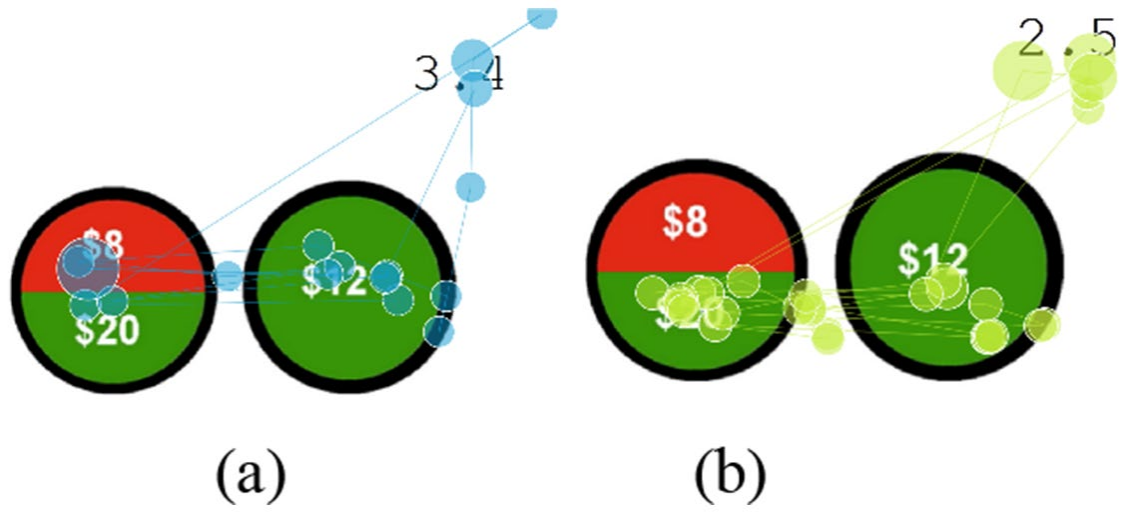
Eye-tracking trajectory plots of safety decision preferences among miners with different levels of emotional exhaustion; (a) miners with low emotional exhaustion; (b) miners with high emotional exhaustion.

Figures 6a,b are eye-tracking heatmaps for miners with low emotional exhaustion, showing shorter gaze times and simpler gaze areas for each type of risk. Figures 6c,d are eye-tracking heatmaps for miners with high emotional exhaustion, indicating longer gaze times and more complex gaze areas for each type of risk.

**Figure 7:**
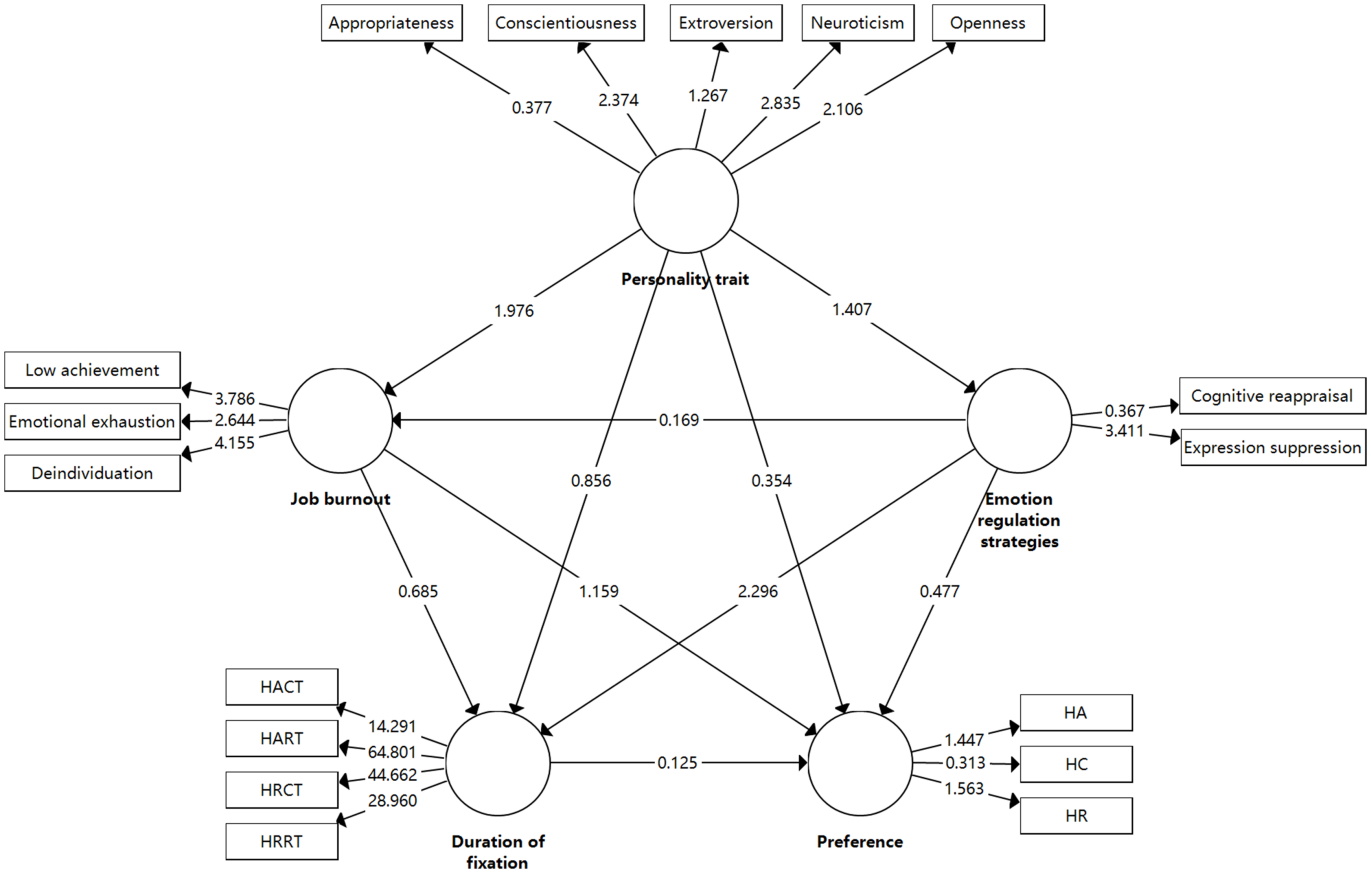
Five-factor PLS-SEM model and path analysis. HACT, A-C gaze duration under time pressure; HART, A-R gaze duration under time pressure; HRCT, R-C gaze duration under time pressure; HRRT, R-R gaze duration under time pressure; HA, Frequency of A-type risk choices under time pressure; HC, Frequency of C-type risk choices under time pressure; HR, Frequency of R-type risk choices under time pressure.

Figures 7a,b respectively depict the eye-tracking trajectory plots of miners with low and high levels of emotional exhaustion. From the figures, it is evident that the eye-tracking trajectories of miners with low exhaustion are clearer, with a greater focus on areas with high saturation in red. Conversely, the eye-tracking trajectories of miners with high exhaustion are more complex, with a greater focus on areas with low saturation in green.

### 4.7 PLS-SEM model and path coefficient test

**Figure 8:**
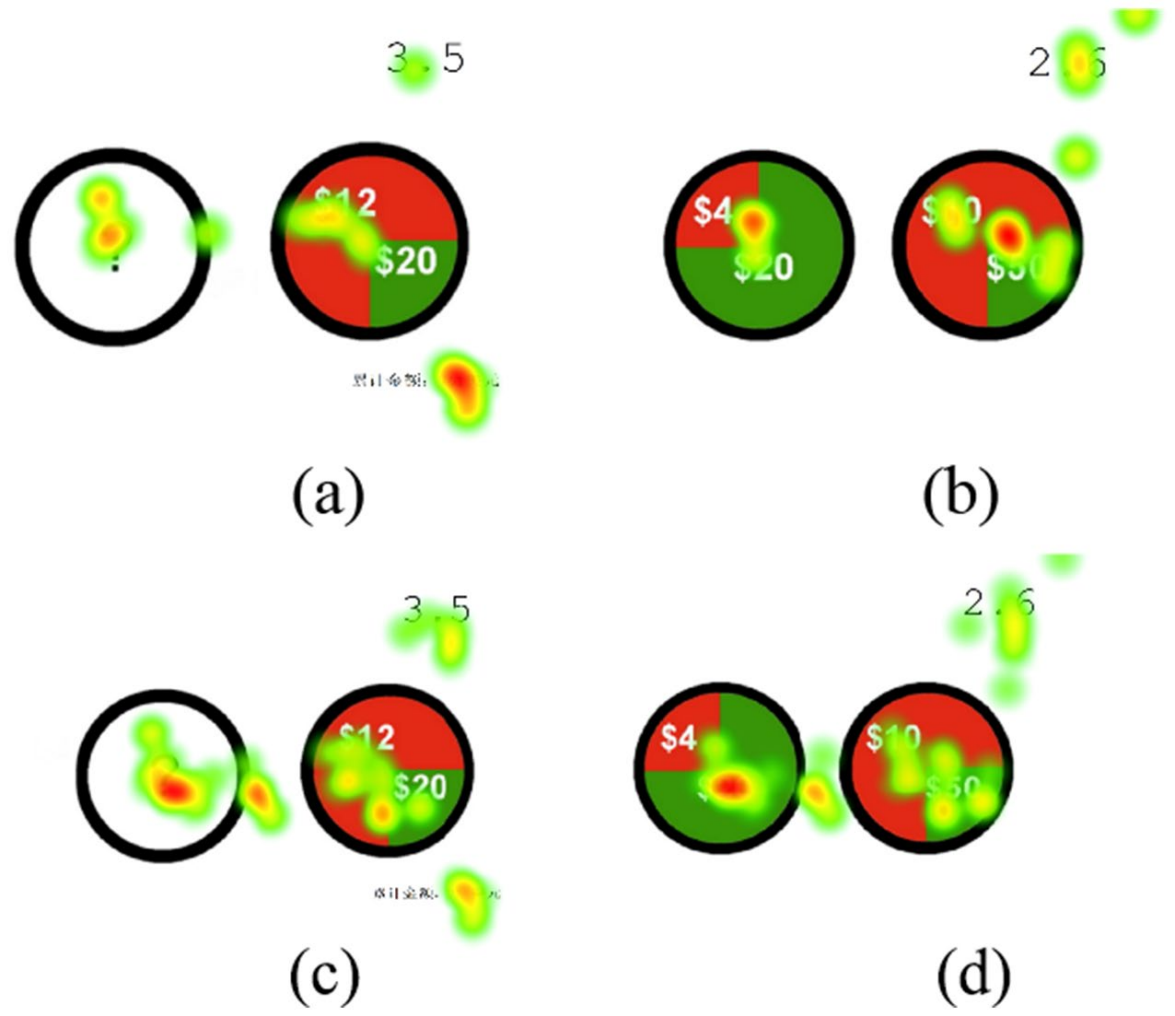
Eye-tracking heatmaps of miners’ safety decision preferences at different levels of emotional exhaustion. (a,b) heatmaps for miners with low emotional exhaustion; (c,d) heatmaps for miners with high emotional exhaustion.

This research employs the PLS-SEM model, which has several advantages over the typical SEM model: (1) This model requires a smaller sample size. (2) It does not require the data to follow a normal distribution for analysis. (3) It can handle complex structural models with multiple dimensions. (4) It can simultaneously handle reflective and formative indicator constructs. (5) It is suitable for theory validation, exploration, and development without requiring a robust theoretical foundation. The PLS-SEM model was constructed with five dimensions of personality traits, two dimensions of emotion regulation strategies, three dimensions of job burnout, gaze duration for four risk combinations, and frequency of three risk selections. Basic bootstrapping was used to test the path coefficients, and the path coefficient diagram is shown in Figure 8.

The total indirect effects of each path are shown in the table below. From Table 9, it can be seen that four paths—personality traits → job burnout, personality traits → emotion regulation strategies, emotion regulation strategies → job burnout, and emotion regulation strategies → gaze duration—pass the significance test.

**Table 9:** Path coefficients test table for total indirect effects in PLS-SEM model.

Pathway	*O*	*M*	STDEV	*T*(|*O*/STDEV|)	*p*
Emotion regulation strategies → gaze duration	0.523	0.451	0.194	2.701	0.010*
Personality traits → job burnout	−0.621	−0.583	0.291	2.134	0.032*
Personality traits → emotion regulation strategies	−0.411	−0.333	0.283	1.452	0.000**
Job burnout → preference for choice	0.498	0.301	0.413	1.204	0.229
Personality traits → gaze duration	0.326	0.235	0.373	0.875	0.382
Job burnout → gaze duration	0.186	0.181	0.26	0.714	0.475
Emotion regulation strategies → preference for choice	0.174	0.139	0.368	0.474	0.636
Personality traits → preference for choice	0.153	0.028	0.411	0.373	0.709
Emotion regulation strategies → job burnout	0.039	0.086	0.22	0.175	0.000**
Gaze duration → preference for choice	0.034	0.077	0.26	0.132	0.895

*Indicates *p* < 0.05, **indicates *p* < 0.01.

## 5 Discussion

### 5.1 Discussion on the relationship between Big Five personality traits, emotion regulation, and job burnout

According to the descriptive statistical analysis and correlation results in Table 10, consistent with previous studies, there is a significant correlation between the Big Five personality traits, emotion regulation, and job burnout.

**Table 10:** Demographic information of the participating miners.

Category	*N*	Age	Percent	Position
Total	48	–	100%	–
Male	48	–	100%	–
Age	23	≤25	47.9%	–
	20	25–35	41.7%	–
	5	≥35	10.4%	–
Associate degree and above	48	–	100%	–
General mining	4 Groups	–	40%	General mining workers
Support	3 Groups	–	30%	Support workers
Electromechanical	3 Groups	–	30%	Electromechanical workers

Regarding the relationship between the Big Five personality traits and job burnout, this study draws the following conclusions: Neuroticism is significantly positively correlated with all three dimensions of job burnout. Individuals with higher levels of neuroticism are more likely to experience job burnout, which is consistent with the findings of Angelini (2023) and Bianchi (2018). Individuals with higher levels of neuroticism mainly exhibit emotional instability, susceptibility to stress, and anxiety. Since the subjects of this study are frontline miners in coal mines, their work is characterized by certain hardships, higher risks, and prolonged physical labor, which increase the physiological and psychological burden on miners. Additionally, miners’ work involves repetitive operations and tasks, lacking variation and challenge. Under these circumstances, individuals with higher levels of neuroticism gradually lose interest and motivation in their work, making them more prone to feelings of exhaustion. Extraversion is significantly negatively correlated with the dimension of emotional exhaustion, a finding also supported by the research results of Wang (2015). Because individuals with higher levels of extraversion enjoy socializing, expressing their feelings, and seeking stimulation, but the standards for miners’ work are very strict, and daily work requirements also demand compliance. Therefore, engaging in repetitive mechanical work for an extended period may lack challenge and stimulation, making it easier for miners with higher levels of extraversion to lose enthusiasm for their work, resulting in physical and mental fatigue. Individuals with higher levels of openness enjoy exploring new things, readily accepting new ideas and experiences. However, the work in coal mines is monotonous, requiring mastery of mature construction knowledge systems, involving repetitive mechanical tasks in daily work, and rotating shift work, which emphasizes rules and procedures more than flexible working hours. This may lead individuals with higher levels of openness to experience less novelty and fewer encounters with new things, making them more likely to lose interest in exploring and learning about their work, leading to a decline in enthusiasm and emotional exhaustion. Individuals with high levels of agreeableness have good adaptability, value maintaining good interpersonal relationships, and can find pleasure and a sense of accomplishment in their work. Therefore, miners with high levels of agreeableness can maintain a positive attitude and derive a sense of accomplishment from their work even when facing heavy and tedious tasks, compared to individuals with significantly different personality traits. At the same time, such miners can actively help prevent and alleviate feelings of job burnout.

In the correlation results between the Big Five personality traits and emotion regulation strategies, this study draws the following conclusions: there is a significant correlation between the Big Five personality traits and the two emotion regulation strategies. Specifically, individuals with high levels of neuroticism or extraversion tend to actively express their emotions and are less likely to suppress their expressions. Individuals with high levels of agreeableness and conscientiousness, on the other hand, tend to choose expression suppression and are more prone to suppressing their emotional expressions. Therefore, the conclusions of this study are consistent with the findings of Shi et al. (2018) and Romosan et al. (2018). Through careful analysis of the measured groups in this study, it is found that for miners with higher levels of neuroticism or extraversion, they are more inclined to actively express their emotions. This means they may be more likely to reveal their emotions at work, including anxiety or stress. While such emotional expression may help them release stress, it may also lead to a tense atmosphere or conflicts in the workplace. However, from another perspective, such miners may not easily suppress their emotions and may also be more adept at identifying and addressing challenges at work. Conversely, miners with higher levels of agreeableness and conscientiousness tend to choose to suppress the expression of emotions. They may place more emphasis on a harmonious work atmosphere and be better at controlling how they express emotions. This implies that they may be calmer and more stable at work, less susceptible to external emotional fluctuations. However, such individuals may also excessively suppress their emotions, leading to long-term accumulation of inner tension and pressure.

In the correlation results between emotion regulation strategies and dimensions of job burnout, this study found the following conclusions: There is a significant correlation between expressive suppression in emotion regulation strategies and the three dimensions of job burnout. Because expressive suppression tends to involve negative emotions, while job burnout arises from individuals engaging in prolonged, repetitive, and monotonous tasks or being unable to fulfill their work duties, resulting in a physiological or psychological adverse state. Therefore, from the causal pathway perspective, if negative emotions generated at work are not adequately expressed but are instead suppressed, the accumulation of these negative emotions may lead to job burnout, especially in dimensions like emotional exhaustion and reduced personal accomplishment. Scholars like Xu et al. (2021) and Lei et al. (2023) have also confirmed this conclusion in their studies. In this study, the reasons why miners using suppression strategies lead to job burnout are multifaceted. Expressive suppression is a psychological mechanism for coping with work pressure and challenges by restraining negative emotions and stress in response to work tasks. However, excessive reliance on suppression strategies over time may lead to psychological fatigue and job burnout. This strategy typically manifests as self-restraint. In the specific occupation of mining, miners face intense physical labor, isolated work environments, and potential safety risks. To cope with these challenges, miners may excessively suppress negative emotions and stress, internalizing feelings of anxiety, frustration, and fatigue without expressing them or seeking support from others. However, prolonged reliance on suppression strategies can have a negative impact on miners’ mental health. Suppressing negative emotions and stress may lead to accumulated psychological pressure, making it difficult for miners to effectively release and cope with inner pressure. This may result in the occurrence of job burnout, manifested as feelings of fatigue, lack of enthusiasm, inability to concentrate, and decreased work quality. It is worth noting that expressive suppression strategies may also hinder effective communication and collaboration among miners. When miners excessively suppress emotions, they may become closed-off, unwilling to share issues, seek help, or communicate with colleagues. This can lead to a tense atmosphere and a sense of isolation in the work environment, further exacerbating the occurrence of job burnout.

### 5.2 Discussion on the mechanism and moderating effect of emotional regulation strategies between personality traits and job burnout

#### 5.2.1 The pathway of conscientiousness → expressive suppression → depersonalization

In exploring the impact of conscientiousness on depersonalization, expressive suppression plays a crucial role as a mediator. Specifically, individuals with high levels of conscientiousness are more likely to cope with external pressures and challenges by controlling and suppressing their expressive behavior. This ability to suppress expression can help individuals maintain stable emotional states and prevent depersonalization. Conscientiousness as an independent variable directly influences depersonalization, suggesting that an increase in conscientiousness reduces the likelihood of depersonalization. However, the effect of conscientiousness on depersonalization is not direct but is mediated through expressive suppression. In practical terms, miners with high conscientiousness often aim to dutifully complete work tasks. Even when experiencing negative emotions, they may choose to suppress their emotional expressions, prioritizing task completion above all else. While this approach may contribute to short-term work continuity and stability, it can lead to negative consequences in the long term. Firstly, excessive expressive suppression may cause individuals to neglect their emotional needs and psychological well-being, thereby affecting their overall welfare. Secondly, prolonged suppression of emotional expression may gradually diminish their ability to establish deep connections with others, resulting in social barriers and communication issues. Personalized expression is one of the key ways people communicate and connect with others. When miners with high levels of conscientiousness are required to suppress personalized expression, they may appear reserved and struggle to articulate their views and emotions in interactions. This not only affects internal team communication and collaboration but may also lead individuals to feel isolated and misunderstood. Individuals who persist in this state over the long term may gradually lose enthusiasm and motivation for their work, leading to a diminished sense of individuality and a detached, perfunctory approach to work, contributing to depersonalized job burnout. Furthermore, excessive suppression of personalized expression may lead to additional adverse outcomes. For instance, it could cause individuals to neglect their own needs, suppress creativity and innovation, and impact job satisfaction and morale. Within teams, excessive expressive suppression might hinder effective information sharing and problem-solving, thereby decreasing overall team performance and efficiency. Moreover, prolonged suppression of expression may leave individuals ill-equipped to cope with stress and challenges effectively, increasing the risk of psychological and physiological health issues.

In conclusion, the relationship between conscientiousness, expressive suppression strategies, and depersonalization is a complex and multidimensional process. Heightened conscientiousness may prompt individuals to adopt expressive suppression strategies to cope with external pressures and challenges. However, excessive use of these strategies can lead to a range of negative outcomes such as social barriers, communication issues, diminished individuality, and job burnout. Therefore, to maintain individual psychological well-being and enhance overall team performance, it is crucial to strike a balance between conscientiousness and expressive suppression. This requires efforts from both individuals and organizational management. Providing effective communication channels, encouraging personalized expression and innovation, and offering psychological support and resources can help individuals better manage stress, maintain a positive mindset, reduce depersonalization, and increase job satisfaction and team cohesion.

#### 5.2.2 Analysis of the moderating effect of expressive suppression between agreeableness and emotional exhaustion

Agreeableness is an important dimension of personality traits, describing the extent to which individuals tend to maintain harmonious relationships with others in social interactions. Individuals with high levels of agreeableness typically show a preference for maintaining good relationships with groups and avoiding conflicts with others. This trait is particularly crucial in work and social environments as it influences how individuals interact with others and manage social conflicts. Individuals with low levels of agreeableness, coupled with low expressive suppression tendencies, tend to prioritize expressing their own emotions. These individuals may influence others through their own affability, thereby reducing their own emotional expenditure. In this scenario, their levels of emotional exhaustion may not be high because they can directly express their feelings and needs, reducing emotional accumulation. However, if these individuals have high expressive suppression tendencies, they may suppress their emotional expression in efforts to make others feel pleasant. While this approach may contribute to short-term social harmony, in the long run, it may lead to higher levels of emotional exhaustion as they continuously suppress their true feelings to meet others’ expectations. Conversely, individuals with high levels of agreeableness typically have higher empathy and are willing to actively resolve conflicts without feeling lost or saddened by suppressing their own emotions. These individuals often manage their emotions better, thereby reducing the risk of emotional exhaustion. However, if highly agreeable individuals have low expressive suppression tendencies, they may be overly direct in emotional expression, potentially leading to increased levels of emotional exhaustion. This is because they may overlook their own emotional needs while constantly meeting the needs and expectations of others.

Expressive suppression plays a crucial moderating role in the relationship between agreeableness and emotional exhaustion. Firstly, expressive suppression can have a positive impact on agreeableness. When individuals effectively employ expressive suppression strategies in social interactions, those with high agreeableness are more likely to exhibit agreeable behaviors and attitudes. For instance, when facing setbacks or conflicts, controlling the expression of emotions can help individuals maintain a calm, rational, and gentle demeanor, facilitating better communication and problem-solving with others. This strategy also allows individuals to consider the feelings of others more when responding, expressing their views appropriately, thus enhancing positive relationships. Secondly, the use of expressive suppression strategies can affect an individual’s level of emotional exhaustion. Excessive or persistent use of expressive suppression may lead to the accumulation and depletion of emotions, increasing the risk of emotional exhaustion. Prolonged suppression of negative emotions in the workplace can leave individuals feeling tired, helpless, and emotionally low, potentially leading to mental and physical health issues. Therefore, employing expressive suppression moderately and flexibly is crucial to avoid excessive emotional suppression and thereby reduce the risk of emotional exhaustion.

In summary, by understanding these relationships and implementing appropriate strategies and measures, individuals and organizations can better manage emotions, enhance the quality of social interactions, and reduce the risk of emotional exhaustion. This not only contributes to individual mental health but also improves team cohesion and work efficiency.

### 5.3 Analysis of safety decision preferences and eye-tracking data among groups with different levels of emotional exhaustion

From the table of equivalent tests, it can be seen that the type A risk that passed the significance test is an ambiguous risk, with the probability and amount of the obtained money unknown, resulting in a 100% ambiguity rate. Miners with low levels of emotional exhaustion are more likely to choose type A risks than those with high levels of emotional exhaustion. From the perspective of cognitive resources, individuals with low levels of emotional exhaustion have more cognitive resources when facing a combination of type 2 risks. They tend to analyze and evaluate various possibilities more calmly when making choices, without relying heavily on intuitive judgments. From the perspective of risk perception, individuals with low levels of exhaustion have higher risk tolerance and coping abilities. This makes them more willing to accept the uncertainty of ambiguous risks and seek potential opportunities. Therefore, individuals with high levels of exhaustion have lower tolerance for uncertainty and tend to choose deterministic or probabilistic risks with known outcomes. This conclusion is consistent with the research conclusion of Huang et al. (2018). From the perspective of emotional state, a positive emotional state may increase individuals’ willingness to take risks. Therefore, individuals with low exhaustion levels have fuller emotional states and are more willing to challenge risky choices. In this decision-making experiment, they tend to choose type A risks with the possibility of higher returns, while individuals with high levels of exhaustion tend to choose conservative returns.

The eye-tracking heatmaps in Figure 8 and eye-tracking trajectory maps in Figure 6 reveal significant differences in the performance of miners with different levels of emotional exhaustion in the risk preference experiment. Firstly, individuals with low emotional exhaustion are often associated with low cognitive load. Therefore, miners with low levels of emotional exhaustion have low levels of cognitive load, resulting in more focused attention and fewer distractions during the observation task. Consequently, their fixation points in the eye-tracking heatmap are more concentrated and their gaze trajectories are clearer and more direct. From a color perspective, the green color scheme has a significant effect on the recovery of negative emotions, making people feel relaxed and calm (Wang et al., 2018). In this experiment, the contrasting colors are red and green, with red carrying more warning and challenge connotations and being easier to detect, while green represents conservatism and safety; for individuals with low exhaustion, their higher cognitive resources will first focus on the red color; whereas individuals with high exhaustion, due to their lower emotional state, tend to choose the conservative green color.

### 5.4 Intervention strategies for miners’ work fatigue

#### 5.4.1 Individual level

From the perspective of miners themselves, understanding their own personality traits and emotional regulation preferences, cultivating a positive mindset, and being able to adjust their emotions reasonably when facing work pressure and challenges are essential. Techniques such as mindfulness and meditation can be used to effectively regulate emotions, enhance work efficiency, and alleviate signs of burnout.

#### 5.4.2 Team level

Promoting teamwork, fostering a supportive and encouraging team atmosphere, enhancing cohesion among team members, and paying attention to miners with different personality traits are essential. Guiding them to adopt appropriate emotional regulation strategies can be helpful. Establishing dedicated psychological support groups to address emotional and job burnout issues among team miners is also beneficial.

#### 5.4.3 Management level

Coal mine management should provide reasonable support and care for miners, promptly address their issues and concerns, and enhance job satisfaction. Establishing effective communication mechanisms to encourage miners to communicate effectively with management is crucial for understanding their real work needs and resources. Providing miners with career development plans and training, ensuring proper job-person fit, can boost their motivation and enthusiasm for work.

#### 5.4.4 Institutional level

Formulating healthy and reasonable work systems, allocating work tasks reasonably to reduce miners’ workload, is essential. Improving compensation and benefits, optimizing reward and punishment mechanisms, enhancing miners’ welfare and security, can strengthen their sense of belonging and loyalty. Strengthening safety measures, focusing on miners’ personal safety, providing adequate safety training and supervision, can reduce work pressure and negative emotions.

## 6 Limitations and prospects

### 6.1 Limitations of the study

This study, based on questionnaire surveys and eye-tracking physiological experiments, investigates the level of work fatigue among miners from the perspectives of personality traits and emotion regulation strategies. There may be certain subjectivity and memory biases in the research process, and respondents’ perception of their own emotion regulation strategies may contain errors, affecting the accuracy of the final results. Additionally, influenced by objective factors, the correlations between individual variables may not be significant, possibly due to the relatively small sample size of the questionnaire study, even though it meets the standards for small-sample research.

### 6.2 Prospects

The experiment of this study was constructed under a profit scenario. Next, the influence of loss scenarios on the results will be explored, as well as the differences between loss and profit scenarios. In future research, new data collection methods will be incorporated on the basis of questionnaire surveys, such as EEG and near-infrared spectroscopy (Tian et al., 2022; Tian et al., 2022), to study the differences in work fatigue among miners from the perspective of cognitive neuroscience. For variables with insignificant correlations, the sample size will be expanded beyond this study, enhancing the generalizability of the research results and comprehensively examining the relationship between miners’ personality traits, emotion regulation strategies, and levels of work fatigue.

## 7 Conclusion

(1) The survey revealed that emotion regulation strategies mediate the relationship between miners’ personality traits and job burnout. Specifically, the expressive suppression strategy fully mediates the relationship between conscientiousness and depersonalization. This finding can help coal mine managers focus on miners with high conscientiousness and address depersonalization burnout caused by expressive suppression by implementing appropriate emotional interventions.
(2) Emotion regulation strategies moderate the relationship between miners’ personality traits and job burnout: the expressive suppression strategy moderates the relationship between agreeableness and emotional exhaustion. This finding helps coal mine managers in developing emotional management training programs, teaching miners how to use emotion regulation strategies more effectively, particularly for those with high agreeableness, to reduce their emotional exhaustion at work.
(3) Understanding miners’ Big Five personality traits and emotion regulation strategies can help analyze the causal paths and manifestations of miners’ job burnout and provide relevant intervention strategies. Organizations can screen employees in a reasonable manner and intervene in miners’ emotion regulation strategies to alleviate negative emotions, which helps to mitigate miners’ job burnout.
(4) Analysis of the results from the safety decision-making preference experiment and the eye-tracking experiment shows that miners with low emotional exhaustion levels are more inclined to choose ambiguous risks compared to those with high emotional exhaustion levels. Their eye-tracking trajectories are clearer, and their heatmaps are more concentrated. The results suggest that miners with low emotional exhaustion levels may prefer ambiguous risks, which could mean they receive sufficient information and emotional support when making safety decisions. Based on this, targeted safety training programs can be developed to improve miners’ risk identification and decision-making abilities. Clear and concentrated eye-tracking trajectories may indicate that miners are more focused and cautious when making decisions. By analyzing eye-tracking data, it is possible to identify miners who may be distracted due to emotional exhaustion and take measures to prevent potential accidents. In terms of job design, the research findings can guide the creation of work environments that better meet miners’ cognitive and emotional needs, reducing errors caused by emotional exhaustion.

## Data Availability

The original contributions presented in the study are included in the article/Supplementary material, further inquiries can be directed to the corresponding author.

## Footnotes

^1^http://www.seecoal.com/plugin.php?id=tom_tctoutiao&mod=info&aid=126

^2^http://www.zjtxy.org.cn/news/xiehui/58801.html
