# Supplementary material for: A multimodal physiological-psychological data-driven study on differentiation in miners’ job burnout and risk preferences
Source: Front Psychol. 2024 Oct 15;15:1438772. doi: 10.3389/fpsyg.2024.1438772 (PMC11519813; doi:10.3389/fpsyg.2024.1438772)
Supplement: Supplementary file 1 [file Table_1.DOCX]

**Appendix**

Questionnaire 1 Big Five personality traits questionnaire

| **Item** | **Fit very well** | **Not quite agree with** | **Some agree with** | **Relatively fit** | **Fit very well** |
| --- | --- | --- | --- | --- | --- |
| 1. I'm not a worrier | 1 | 2 | 3 | 4 | 5 |
| 2. I like having many people with me | 1 | 2 | 3 | 4 | 5 |
| 3. I enjoy immersing myself in fantasies and daydreams and exploring all possibilities and letting them spread and develop | 1 | 2 | 3 | 4 | 5 |
| 4. I try to be polite to everyone I meet | 1 | 2 | 3 | 4 | 5 |
| 5. I put away my things and keep them clean and tidy | 1 | 2 | 3 | 4 | 5 |
| 6. I sometimes feel bitter and indignant | 1 | 2 | 3 | 4 | 5 |
| 7. I love to laugh | 1 | 2 | 3 | 4 | 5 |
| 8. I think it's fun to take up a new hobby | 1 | 2 | 3 | 4 | 5 |
| 9. Sometimes I intimidate or cajole people into doing what I want them to do | 1 | 2 | 3 | 4 | 5 |
| 10. I can arrange my time well and get things done on time | 1 | 2 | 3 | 4 | 5 |
| 11. When I'm in a state of extreme tension, I sometimes feel like I'm going to break down | 1 | 2 | 3 | 4 | 5 |
| 12. I like work that I can do alone without being disturbed by others | 1 | 2 | 3 | 4 | 5 |
| 13. I am fascinated by the expression of art and nature | 1 | 2 | 3 | 4 | 5 |
| 14. Some people think I am selfish and self-centered | 1 | 2 | 3 | 4 | 5 |
| 15. I often encounter situations for which I am not fully prepared | 1 | 2 | 3 | 4 | 5 |
| 16. I seldom feel lonely or depressed | 1 | 2 | 3 | 4 | 5 |
| 17. I like talking to people very much | 1 | 2 | 3 | 4 | 5 |
| 18. I think asking students to listen to controversial people will only confuse their minds and lead them astray | 1 | 2 | 3 | 4 | 5 |
| I am a stubborn, stubborn person | 1 | 2 | 3 | 4 | 5 |
| I will try my best to complete all the tasks assigned to me | 1 | 2 | 3 | 4 | 5 |
| 21, I often feel nervous and extremely uneasy | 1 | 2 | 3 | 4 | 5 |
| I like to join the fun | 1 | 2 | 3 | 4 | 5 |
| Poetry has little or no influence on me | 1 | 2 | 3 | 4 | 5 |
| I know I'm better than most people | 1 | 2 | 3 | 4 | 5 |
| 25. I have a clear set of goals and I am able to achieve them step by step | 1 | 2 | 3 | 4 | 5 |
| Sometimes I feel useless | 1 | 2 | 3 | 4 | 5 |
| 27. I avoid crowds | 1 | 2 | 3 | 4 | 5 |
| Let the mind roam freely is a difficult thing for me | 1 | 2 | 3 | 4 | 5 |
| If I am insulted, I will try to forgive and forget | 1 | 2 | 3 | 4 | 5 |
| 30. It usually takes me a lot of time to settle down to work | 1 | 2 | 3 | 4 | 5 |
| 31. I rarely feel fear or anxiety | 1 | 2 | 3 | 4 | 5 |
| I often feel energetic, full of vitality | 1 | 2 | 3 | 4 | 5 |
| I seldom notice the changes in my mood or feelings in different environments | 1 | 2 | 3 | 4 | 5 |
| I tend to think the best of people | 1 | 2 | 3 | 4 | 5 |
| I work hard in order to achieve my goals | 1 | 2 | 3 | 4 | 5 |
| I am often angry at the way people treat me | 1 | 2 | 3 | 4 | 5 |
| I am a happy and interested person | 1 | 2 | 3 | 4 | 5 |
| 38, I experience many different feelings and emotions | 1 | 2 | 3 | 4 | 5 |
| Some people think I am indifferent, only for themselves | 1 | 2 | 3 | 4 | 5 |
| When I promise to do something, people always believe that I can stick to it | 1 | 2 | 3 | 4 | 5 |
| When things do not go well, I often feel discouraged and want to give up | 1 | 2 | 3 | 4 | 5 |
| 42, Chatting with others does not make me more fun | 1 | 2 | 3 | 4 | 5 |
| 43, reading a poem or viewing a piece of art, sometimes I feel a strong impulse in my heart | 1 | 2 | 3 | 4 | 5 |
| My principle is practical, not emotional | 1 | 2 | 3 | 4 | 5 |
| I should be a reliable and trustworthy person, but sometimes it is not so good | 1 | 2 | 3 | 4 | 5 |
| I am seldom sad or melancholy | 1 | 2 | 3 | 4 | 5 |
| My life is fast-paced | 1 | 2 | 3 | 4 | 5 |
| I have no interest in thinking about the nature of the universe or the present condition of mankind | 1 | 2 | 3 | 4 | 5 |
| 49, Usually I try to be considerate | 1 | 2 | 3 | 4 | 5 |
| I am productive, always able to complete the task in a timely and correct manner | 1 | 2 | 3 | 4 | 5 |
| I often feel helpless and want others to solve my problems | 1 | 2 | 3 | 4 | 5 |
| I am a very active person | 1 | 2 | 3 | 4 | 5 |
| I have a strong curiosity for thinking things | 1 | 2 | 3 | 4 | 5 |
| If I don't like someone, I let them know | 1 | 2 | 3 | 4 | 5 |
| I never seem to be able to do things in order | 1 | 2 | 3 | 4 | 5 |
| 56, Sometimes I feel so ashamed that I almost want to hide | 1 | 2 | 3 | 4 | 5 |
| I would rather act alone than lead others to do it together | 1 | 2 | 3 | 4 | 5 |
| 58. I am often interested in theoretical or abstract concepts | 1 | 2 | 3 | 4 | 5 |
| When necessary, I will manipulate and control others to achieve my purpose | 1 | 2 | 3 | 4 | 5 |
| I try to do everything perfectly | 1 | 2 | 3 | 4 | 5 |

Questionnaire 2: Job Burnout (Mbi-Gs) questionnaire

1. Work exhausts me

○never felt like this0

○Almost no such feeling1

○rarely appears2

○Sometimes appears3

○appears a little frequently4

○appears more frequently5

○Appears every day6

Dealing with colleagues all day at work is really stressful for me ()

○ I've never felt this way0

○ Almost no such feeling 1

○is rarely seen 2

○ Sometimes appears3

○ Slightly frequent 4

○occurs more frequently5

○per day 6

3. Work makes me feel like I'm going to collapse ()

○ I've never felt this way0

○ Almost no such feeling 1

○is rarely seen2

○ Sometimes appears3

○ Slightly frequent 4

○occurs more frequently5

○per day 6

4. Work makes me feel very frustrated ()

○ I've never felt this way0

○ Almost no such feeling 1

○is rarely seen2

○ Sometimes appears3

○ Slightly frequent 4

○occurs more frequently5

○per day6

5. I feel like I'm trying too hard at work ()

○ I've never felt this way0

○ Almost no such feeling 1

○is rarely seen2

○ Sometimes appears3

○ Slightly frequent 4

○occurs more frequently5

○per day6

Working directly with people makes me feel too stressful. ()

○ I've never felt this way0

○ Almost no such feeling 1

○is rarely seen 2

○ Sometimes appears3

○ Slightly frequent 4

○occurs more frequently5

○per day6

7. I feel drained and exhausted by my work

○ I've never felt this way0

○ Almost no such feeling 1

○is rarely seen 2

○ Sometimes appears3

○ Slightly frequent 4

○occurs more frequently5

○per day6

8. I treat my colleagues as emotionless objects

○ I've never felt this way0

○ Almost no such feeling 1

○is rarely seen2

○ Sometimes appears3

○ Slightly frequent 4

○occurs more frequently5

○per day6

When I get up in the morning and have to face the day's work, I feel very tired

○ I've never felt this way0

○ Almost no such feeling 1

○ This feeling is rare 2

○ Sometimes appears3

○ A little frequent 4

○ More frequent occurrence 5

○ Very frequent occurrence 6

I felt that my students and colleagues would blame me for their problems

○ I've never felt this way0

○ Almost no such feeling 1

○ This feeling is rare 2

○ Sometimes appears3

○ A little frequent 4

○ More frequent occurrence 5

○ Very frequent occurrence 6

At the end of the day, I felt that I had lost all patience

○ I've never felt this way0

○ Almost no such feeling 1

○ This feeling is rare 2

○ Sometimes appears3

○ A little frequent 4

○ More frequent occurrence 5

○ Very frequent occurrence 6

12. I don't care what happened to my colleague recently

○ No such feeling 0

○ Almost no such feeling 1

○ This feeling is rare 2

○ Sometimes appears 3

○ A little frequent 4

○ More frequent occurrence 5

○ Very frequent occurrence 6

After taking this job, I have become more and more indifferent to people

○ I've never felt this way0

○ Almost no such feeling 1

○ This feeling is rare 2

○ Sometimes appears3

○ A little frequent 4

○ More frequent occurrence 5

○ Very frequent occurrence 6

14. I feel that this job is gradually making me indifferent

○ I've never felt this way0

○ Almost no such feeling 1

○ This feeling is rare 2

○ Sometimes appears3

○ A little frequent 4

○ More frequent occurrence 5

○ Very frequent occurrence 6

15I have accomplished a lot of meaningful things in my work

○ I've never felt this way0

○ Almost no such feeling 1

○ This feeling is rare 2

○ Sometimes appears3

○ A little frequent 4

○ More frequent occurrence 5

○ Very frequent occurrence 6

16I feel full of energy

○ I've never felt this way0

○ Almost no such feeling 1

○ This feeling is rare 2

○ Sometimes appears3

○ A little frequent 4

○ More frequent occurrence 5

○ Very frequent occurrence 6

17I can easily understand how my colleagues feel

○ I've never felt this way0

○ Almost no such feeling 1

○ This feeling is rare 2

○ Sometimes appears3

○ A little frequent 4

○ More frequent occurrence 5

○ Very frequent occurrence 6

18I can deal with problems brought to me by my colleagues very effectively

○ I've never felt this way0

○ Almost no such feeling 1

○ This feeling is rare 2

○ Sometimes appears3

○ A little frequent 4

○ More frequent occurrence 5

○ Very frequent occurrence 6

19At work, I can handle emotional problems with great composure

○ I've never felt this way0

○ Almost no such feeling 1

○ This feeling is rare 2

○ Sometimes appears3

○ A little frequent 4

○ More frequent occurrence 5

○ Very frequent occurrence 6

20I feel that my work has a positive effect on others

○ I've never felt this way0

○ Almost no such feeling 1

○ This feeling is rare 2

○ Sometimes appears3

○ A little frequent 4

○ More frequent occurrence 5

○ Very frequent occurrence 6

21When I get along with my colleagues, I can easily create a relaxed atmosphere

○ I've never felt this way0

○ Almost no such feeling 1

○ This feeling is rare 2

○ Sometimes appears3

○ A little frequent 4

○ More frequent occurrence 5

○ Very frequent occurrence 6

22I feel happy after working closely with others

○ I've never felt this way0

○ Almost no such feeling 1

○ This feeling is rare 2

○ Sometimes appears3

○ A little frequent 4

○ More frequent occurrence 5

○ Very frequent occurrence 6

Questionnaire 3: Emotional regulation strategy scale

1. When I want to feel something positive (like joy or joy), I change my perspective

Strongly disagree.1 2 3 4 5 6 7 strongly agree

2. I don't show my emotions

Strongly disagree. 1 2 3 4 5 6 7 strongly agree

3. When I want to feel less negative emotions (like sadness or anger), I change my perspective

Strongly disagree. 1 2 3 4 5 6 7 strongly agree

4. When I feel positive emotions, I am very careful not to show them

Strongly disagree. 1 2 3 4 5 6 7 strongly agree

5. When faced with a stressful situation, I make myself think about it in a way that helps me stay calm

Strongly disagree. 1 2 3 4 5 6 7strongly agree

6. The way I control my emotions is by not expressing them

Strongly disagree. 1 2 3 4 5 6 7 strongly agree

7. When I want to feel more positive emotions, I change the way I think about the situation

Strongly disagree. 1 2 3 4 5 6 7 strongly agree

8. I control my emotions by changing the way I think about situations

Strongly disagree. 1 2 3 4 5 6 7strongly agree

9. When I feel negative emotions, I make sure I don't show them

Strongly disagree. 1 2 3 4 5 6 7 strongly agree

10. When I want to feel less negative emotions, I change the way I think about the situation

Strongly disagree. 1 2 3 4 5 6 7 strongly agree
